# Application of retinoic acid improves form and function of tissue engineered corneal construct

**DOI:** 10.1080/15476278.2015.1093267

**Published:** 2015-10-23

**Authors:** Fadhilah Z Abidin, Ricardo M Gouveia, Che J Connon

**Affiliations:** Institute of Genetic Medicine; Newcastle University; Center for Life; Newcastle, UK

**Keywords:** 3D model, collagen gel, Cornea, in vitro, keratocytes, retinoic acid, tissue engineering

## Abstract

Retinoic acid has recently been shown to control the phenotype and extracellular matrix composition of corneal stromal cells cultured *in vitro* as monolayers. This study set out to investigate the effects of retinoic acid on human corneal keratocytes within a 3D environment. Human corneal keratocytes were encapsulated in collagen gels, which were subsequently compressed under load, and cultured in serum-free media supplemented with 10 µM retinoic acid or DMSO vehicle for 30 days. Cell proliferation was quantified on selected days, while the expression of several important keratocytes markers was evaluated at day 30 using RT-PCR and immunoblotting. The weight and size of the collagen constructs were measured before and after hydration and contraction analyses. Retinoic acid enhanced keratocyte proliferation until day 30, whereas cells in control culture conditions showed reduced numbers after day 21. Both gene and protein expressions of keratocyte-characteristic proteoglycans (keratocan, lumican and decorin), corneal crystallins and collagen type I and V were significantly increased following retinoic acid supplementation. Retinoic acid also significantly reduced the expression of matrix metalloproteases 1, 3 and 9 while not increasing α-smooth muscle actin and fibronectin expression. Furthermore, these effects were also correlated with the ability of retinoic acid to significantly inhibit the contractility of keratocytes while allowing the build-up of corneal stromal extracellular matrix within the 3D constructs. Thus, retinoic acid supplementation represents a promising strategy to improve the phenotype of 3D-cultured keratocytes, and their usefulness as a model of corneal stroma for corneal biology and regenerative medicine applications.

## INTRODUCTION

The cornea is a transparent, avascular structure that provides optical transparency for light transmission and light refraction for normal vision as well as protection against external environment.^1,2^ The World Health Organization has estimated over 10 million people from around the world suffer from corneal blindness secondary to trauma, infection or hereditary diseases.^3^ Although transplantation is the prevailing option to correct severe corneal impairment, the sustainability of this treatment of choice remains a great challenge. This is mainly due to limited supply of corneas from healthy corneal donors, and is made worse by the growth in refractive surgeries, which degrade the cornea for subsequent use in transplantation.^4^ These issues have fueled interest in the development of tissue-engineered corneas as a long-term strategy to address the aforementioned problems.^5,6^ In this context, natural biological materials such as collagen, has been used to provide scaffolding support for corneal tissue engineering with promising results.^7-9^

Collagens are one of the most abundant structural proteins within mammalian connective tissue and represent almost one-third of total body proteins.^10^ Collagen provides structural support and mechanical stability while maintaining flexibility in various tissues, such as skin,^11^ tendon,^12^ bone.^13^ and cornea.^14^ Collagen type-I is one of the main structural elements of the extracellular matrix of the corneal stroma. As such, its use as a corneal tissue engineering scaffold has been extensively studied.^15-18^ The use of collagen in tissue engineering has been attributed to its biocompatibility, biodegradability and low-immunogenicity.^19^ However, conventional collagen gels are highly hydrated and therefore, difficult to be manipulated.^20^ An additional step of ‘plastic compression’ following collagen gel formation allows the controlled engineering of collagen gels by rapid dehydration, and subsequently causing permanent deformation and a marked increase in collagen fibril density.^21,22^ Compressed collagen gels are mechanically robust structures comprised of compacted collagen fibrils with a smooth surface topography.^23^ It has been suggested that compressed collagen gels are a suitable mimic for the structure of biological materials,^21^ and several studies have used corneal keratocytes in the collagen scaffold to produce a 3D tissue culture to mimic the presence of keratocytes in normal corneal stroma.^24-28^

Keratocytes are mesenchymal-derived, flattened cells which sparsely populate the corneal stroma.^29,30^ Their primary function is to synthesize and maintain the extracellular matrix (ECM) component of the stroma that comprises the structural backbone of the cornea.^31^ Keratocytes are quiescent in nature and exhibit slow turn over.^32^ The *ex vivo* expansion of keratocytes has been performed in order to investigate its biology *in vitro*, but maintaining them in their quiescent phenotype remains a considerable challenge. The use of serum-free media was shown to maintain the quiescence phenotype, but not support proliferation, of corneal stromal cells,^33-35^ thus making cultivation of large quantities of cells by subculturing a difficult task. On the other hand, the addition of serum to culture medium helps to accelerate cell proliferation, but at the same time results in the differentiation of keratocytes to fibroblasts with altered physiological and biochemical properties, namely its phenotype, keratan sulfate production and the expressions of certain genes such as α-smooth muscle actin, aldehyde dehydrogenase (ALDH) and fibronectin.^29,34,36^ Previous studies have tested several media formulations that allow corneal stromal cells to proliferate and survive for extended period in culture while maintaining the expression of keratocyte-specific markers. In particular, fibroblast growth factor 1 (FGF-1),^37^ FGF-2,^38^ FGF-2 + heparin,^37^ and transforming growth factor β3 (TGF-β3)^39^ have been used to reverse the serum-induced (myo)fibroblast transformation. Therefore, the challenge now is to find a new supplement for serum-free media that allows cell growth and proliferation without encouraging fibroblastic differentiation.

One of the possible supplements for serum-free growth of keratocytes is retinoic acid (RA), a molecule that plays a crucial role in vertebrate development, cellular differentiation and homeostasis,^40-42^ and is essential to ocular health.^43-45^

The human body produces RA, an active form of vitamin A, through two sequential oxidation steps: from retinol (absorbed by intestinal mucosa cells following dietary ingestion) to retinaldehyde, and then to RA.^46^ Its use has been implicated in cutaneous wound healing,^47-49^ and considerable interest has been steered towards its application in ophthalmology. As such, much of the current lines of research focuses on its potential use in improving corneal wound healing through faster epithelialization,^50-52^ however, little is known about the effect RA has on corneal keratocytes in their native 3D environment.

In this study, we sought to investigate the effects of RA supplementation in serum-free medium on corneal keratocytes in a 3D environment for tissue engineering purposes. We considered its effect on cell proliferation, collagen production, hydration of tissue constructs and the expression of keratocytes markers at both transcriptional and protein levels in an extended period of culture.

## RESULTS

### Effects of RA on keratocytes proliferation

**FIGURE 1.**
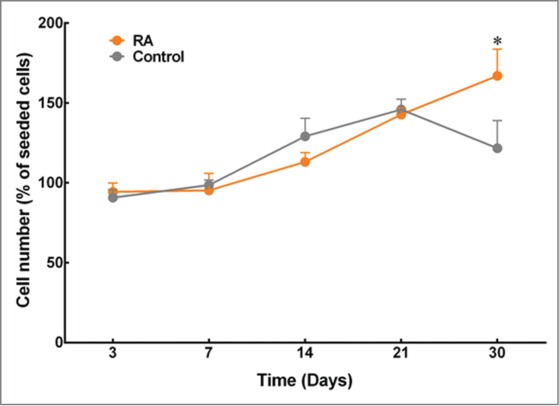
Effects of RA supplementation on the proliferation of keratocytes embedded in compressed collagen gels. Quantification was performed using AlamarBlue® assay, and cell numbers were normalized as a percentage of cells initially seeded. Data (mean ± SD) were obtained from 3 independent experiments (*n* = 3) and compared using *t*-test (*corresponds to *P* < 0.05).

The proliferation assays showed a steady increase in the number of cells from day 3 to day 30 in the RA-supplemented group and from day 3 to day 21 in the control group (**Fig. 1**). The cell numbers of keratocytes in collagen gels cultured using medium with or without supplementation of RA showed comparable values up to 21 d in culture. However, keratocytes cultured in medium supplemented with RA continued to proliferate until day 30, whereas keratocytes cultured in control medium showed a significant decline in cell number. Hence, day 30 was taken as the day for further assessment on the expression of keratocytes markers.

### Effects of RA on engineered tissue contraction assay

**FIGURE 2.**
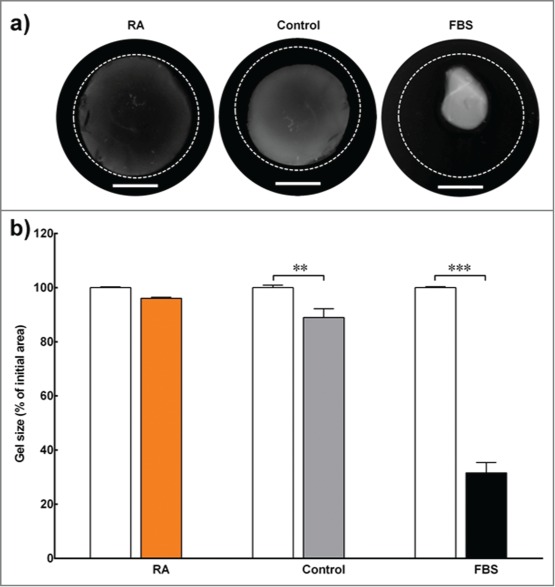
Contraction assay. (**A**) Contraction assay of 3D collagen constructs embedded with corneal keratocytes and cultured in serum-free medium (with RA supplementation or DMSO control) or standard medium (with serum; FBS). The outer dotted line represents the size of the gel at the beginning of the experiment. Scale bar = 10 mm. (**B**) The gel size (in percentage) was determined by calculating the difference in constructs' surface area between the initial day of experiment (white bar) and day 30 (colored bar). Data (mean ± SD) were obtained from 3 independent experiments (*n* = 3) and compared using *t*-test (** and *** correspond to *P* < 0.01 and <0.001, respectively).

We used digital images of the engineered stromal constructs to calculate the percentage change in surface area relative to their initial surface area following 30 d of culture (**Fig. 2A**). The engineered stromal constructs cultured in RA-supplemented medium successfully maintained more than 95% of their surface area at the end of the experiment (**Fig. 2B**). On the other hand, the surface area of the constructs cultured in serum-free medium with DMSO (control) were reduced by more than 20% of their original surface area, and the constructs cultured with serum (FBS) contracted more than 60% of their initial surface area. The differences in gel size within the control and FBS groups were significant for the duration of the experiment (p < 0 .01 and < 0 .001, respectively).

### Effects of RA on engineered tissue hydration

**FIGURE 3.**
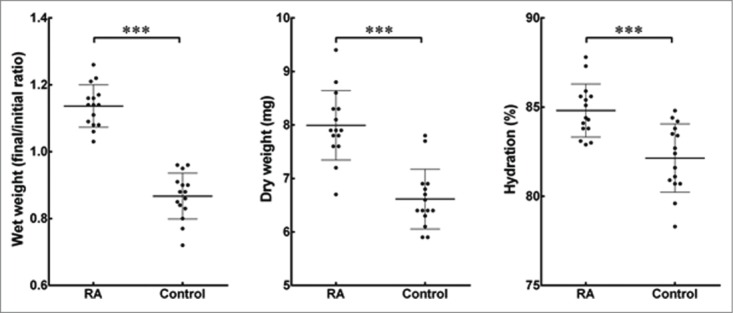
Effects of RA supplementation on the hydration of cell-encapsulating compressed collagen gels. The weight of all gels was measured before and after the 30 d culture period to find the differences in the wet weight. The dry weight was quantified following freeze-drying of the gels. The percentage of hydration denotes the loss of bound water from the collagen gels with or without supplementation of RA. Data (mean ± SD) were obtained from 3 independent experiments (*n* = 3) and compared using *t*-test (*** correspond to *P* <0.001).

Interestingly, there was a significant increase in the wet weight of collagen gels containing keratocytes supplemented with RA as compared to the control after 30 d (**Fig. 3, left panel**). Not only that, the results also showed that collagen gels in the control groups reduced their initial weight following the 30-day culture period. The dry weight of freeze-dried gels from the RA-supplemented group was also found to be significantly higher by 18% than the controls (**Fig. 3, central panel**). At the same time, the hydration percentage within RA-supplemented collagen gels showed to be higher compared to the collagen gels from the control group (84 ± 1 and 80 ± 2%, respectively; **Fig. 3, right panel**).

### Effects of RA on the expression of keratocytes markers

**FIGURE 4.**
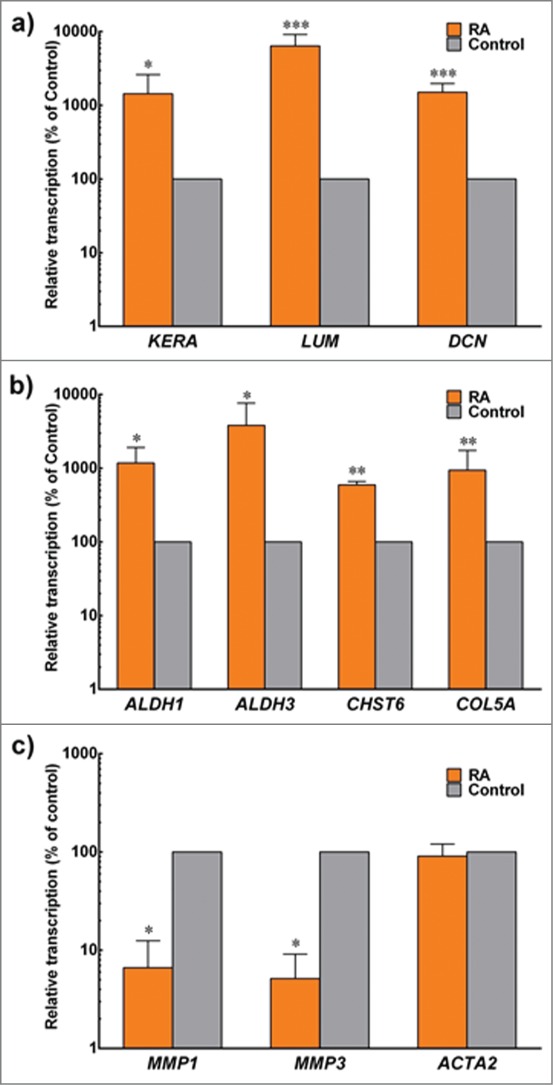
Expression of keratocyte markers at the transcriptional level. Total mRNA from cells embedded in compressed collagen gels cultured for 30 d in control (gray) and +RA (orange) was extracted and analyzed by RT-PCR. Gene expression was normalized relative to that of control group. Data (mean ± SD) were obtained from 3 independent experiments (*n* = 3) and compared using *t*-test (*, ** and *** correspond to *P* < 0.05, <0.01 and <0.001, respectively).

Several important keratocytes markers were analyzed at the gene transcript level and compared with the control. Results showed that RA treatment significantly increased transcription of genes coding for key corneal proteoglycans (keratocan, lumican and decorin) by 1436 ± 12, 6410 ± 37, and 1510 ± 5 % of the control, respectively (**Fig. 4**). In addition, transcription levels of genes coding for corneal crystallins (ALDH1 and ALDH3), CHST6 and Collagen type V were also studied and shown to increase by 1181 ± 7, 3826 ± 38, 596 ± 1 and 941 ± 8 % of the control, respectively. On the other hand, genes coding for two keratocytes matrix metalloproteases, MMP1 and MMP9 were significantly down regulated to 6.6 ± 0.04 and 5.2 ± 0.04 %, respectively, when compared to the control (**Fig. 4**). No difference was observed for transcripts of *ACTA2*, the gene coding for αSMA.

**FIGURE 5.**
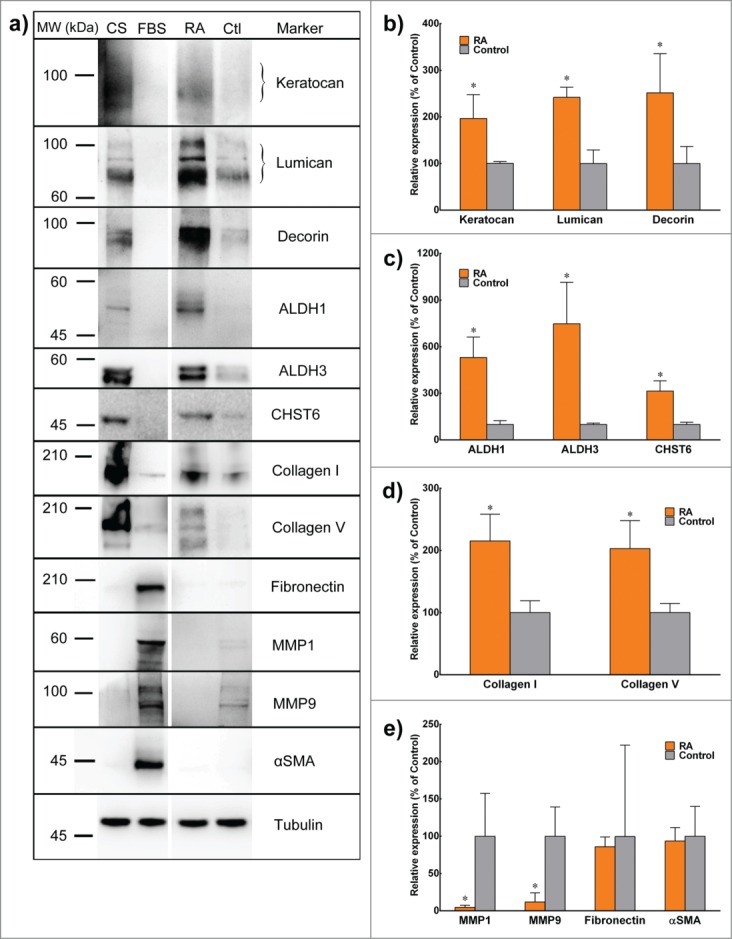
Expression of keratocyte markers at the protein level. (**A**) Lysates from corneal stroma (CS), corneal fibroblasts cultured in FBS (FBS), or 3D stromal constructs supplemented with RA (RA) or DMSO (Ctl) were extracted and analyzed by reducing SDS-PAGE followed by immunoblotting. (**B-E**) Quantification of protein expression from control (gray) and RA (orange) was performed by immunoblot densitometry for all markers. Protein expression was normalized relatively to that of control group. Data (mean ± SD) were obtained from 3 independent experiments (*n* = 3) and compared using *t*-test (* correspond to *P* < 0.05 respectively).

Furthermore, these data were supported by protein expression levels analyzed by immunoblotting and quantified by densitometry. RA-supplemented keratocytes showed significant percentage-increased expression for keratocan (197.4 ± 0.5), lumican (251.7 ± 0.5), decorin (321.2 ± 0.5), Collagen type I (198.5 ± 0.3), Collagen type V (210.4 ± 0.8), ALDH1 (575.9 ± 2.9), ALDH3 (301.2 ± 0.6) and CHST6 (689.0 ± 3.5) relative to the control (**Fig. 5**). Again similar to gene transcript levels, the expression of MMP-1 and MMP-3 in RA supplemented group was significantly reduced to 7.4 ± 0.08 and 10.1 ± 0.07 % of the control levels, respectively (**Fig. 5**). In addition, αSMA and fibronectin levels were not affected by RA supplementation, and remained residual compared with the heightened levels normally seen in FBS-activated fibroblasts.

**FIGURE 6.**
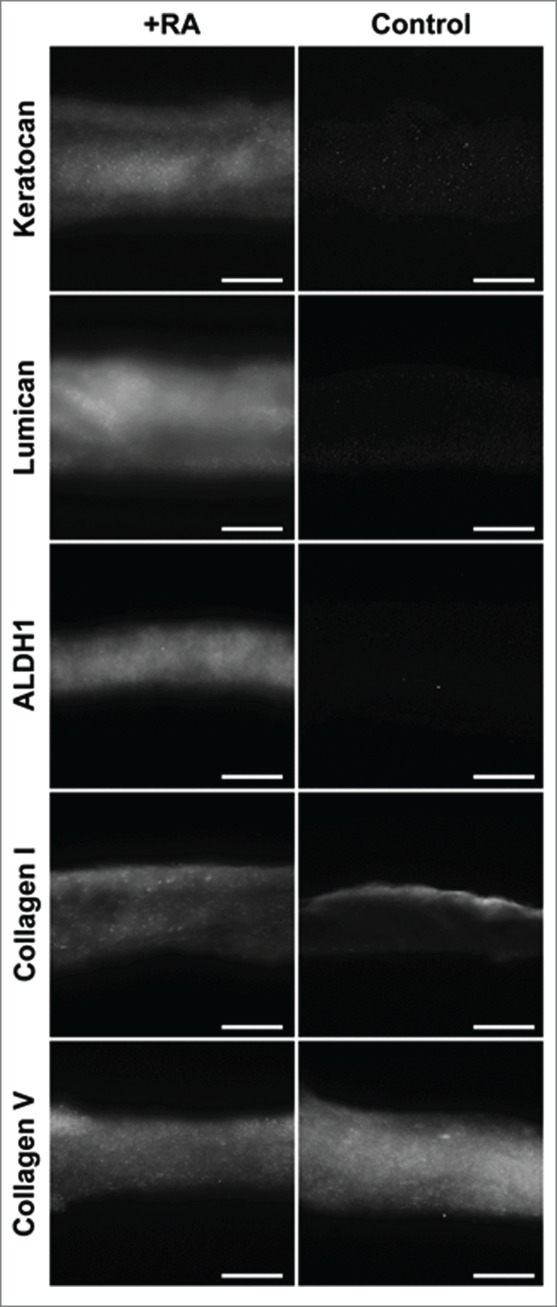
Immunofluorescence staining of ECM proteins characteristic of the corneal stroma, expressed by encapsulated keratocytes within compressed collagen gels with or without RA supplementation. All photographs were taken at x200 magnification. Scale bar = 50 µm.

The localizations of these proteins, shown by immunofluorescence analysis, were not substantially modified by RA supplementation despite their stronger detections. However, localization of Collagen type V was shown to be similar throughout the depth of collagen gels in both groups despite its stronger detection in the RA-supplemented group at both transcriptional and protein levels (**Fig. 6**)

.

## DISCUSSION

Our previous work has shown that using an optimal concentration of RA encouraged the keratocytes to grow and proliferate further without the need for serum in the medium.^53^ In this study, we extended the experiment into a 3D construct to investigate the use of RA supplementation in the generation of potential biologically relevant tissue-engineered corneal stroma. The use of RA for *in vitro* keratocytes studies holds great promise, since we have shown that its supplementation successfully induced proliferative potential of keratocytes when used in serum-free medium over an extended period in culture. This is important to tissue engineering as engineered constructs, such as the cornea, typically require many days in culture.

In the present study, we demonstrated that supplementation of RA in serum-free medium to culture corneal fibroblasts in a 3D collagen construct helped prevent cells from becoming fibroblastic and remained more keratocyte-like as evidenced by an increasing production of keratocyte-markers and very low expressions of fibroblastic-markers. Chemical cues are known as important elements in the control and maintenance of the keratocyte phenotype.^35^ A considerable challenge in stromal cell culture is to encourage the keratocytes to proliferate while at the same time maintaining the keratocyte phenotype in order to continue producing the ECM proteins essential for optical transparency. Previous attempts have been made towards obtaining a proliferating culture of ‘pure’ keratocytes, including supplementation with ascorbic acid,^54^ insulin,^55^ growth factors,^56^ cytokines,^57^ and using low glucose media.^58^ Within the 3D environment, we found that RA significantly modulated the expression of many keratocyte-characteristic ECM components (keratocan, lumican, decorin), the corneal crystallins (ALDH1, ALDH3) and carbohydrate sulfotransferase 6 (CHST6), as well as increased expression of both Collagen type I and V.

Keratocytes secrete Collagen type I and V, which are the predominant fibrillar collagens in the corneal stroma. In addition, the uniformity of these fibrillar collagens (diameter and interfibrillar spacing) is important for the maintenance of corneal function. Keratocan, lumican and decorin belong to the family of small leucine-rich proteoglycans (SLRPs) which serve as regulators of tissue hydration and collagen fibrillogenesis.^59,60^ These SLRPs bind to fibrillar collagens and affect the collagen matrix assembly needed for corneal transparency.^61^ Furthermore, significant increases in ALDH1 and ALDH3 expression within the RA-supplemented group were also important findings. Prominent ALDH isoenzymes in cornea, such as ALDH1 and ALDH3, exert protective effects from the harmful effects of UV-induced lipid peroxidation,^62-64^ and maintain corneal transparency.^65^ ALDH is produced in greater amounts by quiescent keratocytes compared to their activated phenotype, as shown both *in vitro* (following exposure to serum)^66,67^ and *in vivo*.^68^ As such, a decrease in corneal crystallins is posited to increase light scattering by the corneal fibroblasts, and decrease corneal transparency.^69^ Interestingly, RA significantly modulated the expression of CHST6, an enzyme that catalyzes the transfer of sulfate group to keratan sulfate proteoglycans (KSPGs) to help maintain corneal transparency. CHST6 is mainly expressed by the keratocytes both *in vivo* and *in vitro.*^70^ Defects in this gene are associated with macular corneal dystrophy (MCD), a rare autosomal recessive inherited disorder characterized by abnormal deposits of unsulfated KSPGs that causes progressive stromal haziness leading to visual impairment.^71,72^ An increase synthesis of CHST6 following RA supplementation suggests that RA is safe for corneal keratocytes. At the same time, this result implies the considerable potential for RA to be used as a possible therapy for MCD, as the aim in curing this rare genetic disorder must involve and target corneal keratocytes.^70^

The expression of MMP1 (i.e. interstitial collagenase) and MMP3 (i.e. stromelysin-1) was greatly reduced following RA supplementation, in line with previous findings^73^ in diabetic human skin, which showed enhanced collagen synthesis and reduction in MMP expression in the presence of RA. Under normal circumstances, MMP-1 and MMP-3 are not synthesized by quiescent keratocytes. However, the synthesis is upregulated when the keratocyte's phenotype changes into that of repair fibroblasts.^74,75^ We also evaluated the expression of α-SMA and fibronectin and found that they appear to be unaffected by the presence of RA in the cultures i.e. low, as one would expect from serum-free conditions.

The increase in ECM production following RA-supplementation may also contribute to the measured increase in both wet and dry weight of the 3D collagen-keratocyte constructs. The wet weight of gels significantly increased after the 30-days period in culture in RA conditions compared to their initial wet weight, but decreased in control gels, indicating that RA induced the resident keratocytes to 1) produce larger amounts of newly-synthesized material, 2) enhanced the gels' ability to accumulate water, and/or 3) inhibited degradation of the initial collagen gel scaffold compared to control conditions. These conclusions are supported by the significant increase in dry weight of RA-treated gels (**Fig. 3, central panel**), as well as by the RA-induced enhancement of keratocyte-characteristic ECM components and the abrogation of MMP expression observed at the gene and protein levels (**Fig. 4 and 5** respectively). Moreover, the higher content of keratocyte-characteristic proteoglycans (keratocan, lumican and decorin) in the RA-treated group might account for the higher capacity of these gels to retain water (**Fig. 3, right panel**). As mentioned previously, the SLRPs serve as regulators for tissue hydration. Since there were more SLRPs in collagen constructs supplemented with RA, it was not surprising that the water content within these gels was higher compared to the control. Therefore, both wet and dry weight values were important to understand the dynamic changes in the properties in this gel-keratocyte system, and were necessary to determine the constructs' hydration, an important property of the corneal stroma and its transparency.

Importantly, the contraction assay confirmed that the addition of RA significantly reduced contraction of engineered constructs compared to the control gels. This is another positive finding showing that RA was not only mitogenic, but also maintained keratocytes in a non-contractile phenotype. However, the potentially limiting effects of increased collagen (as shown in the presence of RA) on gel contraction cannot be disregarded. The use of fetal bovine serum in the culture medium to promote cell proliferation results in contraction of the engineered matrix^76^ that often mimic scarred native tissue due to activation of stromal fibroblasts into myofibroblasts.^77^ While removal of serum can abrogate this phenomenon, we have demonstrated that further significant loss of contraction can be achieved by the simple addition of RA.

## MATERIALS AND METHODS

### Human corneal keratocyte isolation

Corneal tissues were obtained from Royal Berkshire Hospital, Reading, UK and Royal Victoria Infirmary, Newcastle, UK. Corneal stroma was excised from cadaverous human limbal tissue following removal of the central 7 mm for keratectomy (donors' age between 39 and 76; average ± SD = 61 ± 12 years, with no prior history of corneal diseases or ocular trauma). The male-female donor ratio was 2:3. Briefly, the epithelia-depleted corneal tissues were minced using a scalpel, transferred to DMEM:F12 medium (Invitrogen, 31331–028) supplemented with 5% fetal bovine serum (FBS; Gibco Invitrogen, 10082–147), 2 g/L (450 units/ml) collagenase type-1 (Invitrogen, 17018–029) and incubated at 37°C under continuous rotation for 5 hours, followed by incubation with 0.25% trypsin-EDTA (Gibco Invitrogen, 25200–072) for 10 minutes. The isolated keratocytes were plated onto tissue culture flasks (Greiner Bio-One, 658175) and maintained using culture medium containing DMEM:F12 medium (Invitrogen), 5% FBS (Gibco Invitrogen), 1 × 10^−3^ M ascorbic acid (Sigma-Aldrich, A8960), 1 × ITS liquid media supplement (Sigma-Aldrich, I3146) and 1% penicillin/streptomycin (Invitrogen, 15140–122). The culture medium was changed every 2–3 days and cultures were maintained until reaching 70–80% confluence. Cells were then transferred to culture medium without FBS (serum-free medium, SFM) to induce quiescence, and passaged after 3 days. Corneal stromal fibroblasts maintained in serum-containing media have been previously shown^56-58^ to revert to a more keratocyte-characteristic phenotype, expressing higher keratocyte-specific markers^58^ while showing reduced cell migration and contractility after a 3-day period of serum starvation.^56-58,78^ Each subsequent experiment was performed 3 independent times using keratocytes from passages 3 to 5 from specific donors.

### Formation of compressed collagen gels with embedded keratocytes

Collagen gels were prepared as previously described.^79^ 2 ml sterile rat-tail collagen type I (2.2 mg/ml in 0.6% acetic acid, First Link Ltd.,60–30–810) and 0.5 ml modified Eagle's minimum essential medium (MEM; Fisher Scientific, 21430–020) were mixed in a centrifuge tube placed on ice. Sodium hydroxide 1 M (Fisher Scientific, J/7620/15) was added drop-wise to neutralize the pH of the mixture, along with intermittent, gentle mixing of the solution until it changed its color from yellow to light pink. The mixture was then cast into 12-well plate molds (Greiner Bio-One, 665180), along with 2.5 × 10^5^ serum-deprived keratocytes in each well prior to polymerization (gelling), which subsequently took place for 30 minutes at 37°C in a humidified 5% CO_2_ incubator. Next, the polymerised collagen gels containing keratocytes were removed from the molds and placed in between layers of nylon mesh, and the compression was performed at room temperature using a 134 g load for 5 minutes. The resulting compressed collagen gels embedded with keratocytes were then transferred into 6-well plates (Greiner Bio-One, 657160) and cultured using SFM supplemented with either 1 × 10^−7^ M RA (+RA, Sigma-Aldrich, R2625) or equivalent volume of DMSO vehicle (control; Fisher Scientific, 10080110) for 30 days. The culture medium was changed every 2–3 days.

### Proliferation assay

The AlamarBlue® assay was used to assess keratocyte proliferation and viability within the collagen gels with or without supplementation of RA in SFM for 30 days. The gels were incubated with resazurin reagent (Sigma-Aldrich, R7017; prepared in 1:10 dilution using fresh culture medium) for 2 h at 37°C, after which 100 µl of culture supernatants were sampled in triplicate for fluorescence emission analysis at 590 nm using Fluoroskan Ascent fluorescent spectrophotometer (Thermo Labsystems, Franklin, MA), and cells replenished with fresh media. The assay was done on days 3, 7, 21 and 30 of culture. Cell number was calculated by interpolation using a standard curve for fluorescence values of 1, 5, 10, 20, 50, and 100 × 10^4^ cells, and the values correspond to average ± SD of 3 independent experiments.

### Hydration study

The weight of all cell-encapsulated collagen gels from RA-supplemented and control groups was measured before starting the experiment to find the initial weight (W_i_), and similarly after the 30-day culture period for the final weight (W_f_). The ratio of weight increment (wet weight) was then calculated using the following equation (Eq 1).

(1) $$\begin{array}{l} \text{Wet weight}\left(\text{ratio}\right)=\text{final weight}\left({\text{W}}_{\text{f}}\right)\\ /\text{initial weight}\left({\text{W}}_{i}\right) \end{array}$$

Next, we used a freeze-drying method to sublimate frozen water from the solid to gas phase under controlled pressure in order to determine the percentage of water bound to these gels. The weight of all freeze-dried gels were again measured to find the dry weight (W_d_), which was later used to find the hydration percentage of all gels using the following equation (Eq 2).

(2) $$\text{Hydration}\left(\%\right)=\left({\text{W}}_{\text{f}}-{\text{W}}_{\text{d}}/{\text{W}}_{\text{f}}\right)\times 100$$

This experiment was performed 3 independent times, using 3 replicate gels. 

### Gel contraction assay

Digital photography was used to image the constructs at the beginning of the experiment and on day 30 of culture. The images were then analyzed using Image J v1.46 (National Institute of Health, Bethesda, MD). For each experiment, 3 collagen gels containing keratocytes which were either cultured in serum-free medium (with RA or DMSO) or standard culture medium with serum (FBS) were evaluated and the average contraction was determined by calculating the percent decrease in surface area compared to the original gel surface area.

### Reverse Transcription Polymerase Chain Reaction (RT-PCR) analysis

Following 30 days in culture, the compressed collagen gels with embedded keratocytes were harvested, and RNA was isolated using standard Trizol (Invitrogen, 15596–018) extraction. The assessment of RNA quality was performed using Nanodrop 2000 spectrophotometer (Thermo Scientific, UK) to ensure the 260/280 ratio was within the range of 1.7 to 2.0. Synthesis of cDNA from isolated total RNA was done using the RT2 First Strand kit (Qiagen, 330401) according to the manufacturer's protocol, in a TcPlus thermocycler (Techne, Staffordshire, UK). The polymerase chain reaction (PCR) was carried out using the default thermal profile of the Eco Real-Time System (Illumina, San Diego, CA), with the following 40 × 3-step cycle: 10-second denaturation, 95°C; 30-second annealing, 60°C; 15-second elongation, 72°C. The relative expression of keratocyte genes coding (summarized in **Table 1**) were calculated by the comparative threshold cycle (CT) (Eco Software v3.1; Illumina) and normalized to the expression of the *POLR2A* housekeeping gene. Results from 3 independent experiments from 3 different donors were normalized relative to the expression from compressed collagen gels embedded with keratocytes cultured using control media.

**TABLE 1.** Description of primers used in RT-PCR for keratocyte gene expression analysis

Gene	Primers 5′→3′
*POLR2A* (housekeeping gene)	F: cat cat cga aga caa tgg tgR: aac aat gtc ccc atc aca ca
*Keratocan (KERA)*	F: tat tcc tgg aag gca agg tg R: acc tgc ctc aca ctt cta gac c
*Lumican (LUM)*	F: cct ggt tga gct gga tct gt R: tag gat ggc ccc agg a
*Decorin (DCN)*	F: aat tga aaa tgg ggc ttt cc R: ctg ctg att ttg ttg cca tc
*Aldehyde dehydrogenase 1 (ALDH1)*	F: ctc tca ctg ctc tcc acg tg R: gag aag aaa tgg ctg ccc ct
*Aldehyde dehydrogenase 3 (ALDH3)*	F: ccc ctt caa cct cac cat cc R: gtt ctc act cag ctc cga gg
*Carbohydrate sulfotransferase 6 (CHST6)*	F: acg acg ttt ggg agc ctt t R: tag agg ttc ctc agc acc cca
*Collagen Type V (COL5A)*	F: atc ttc caa agg ccc gga tg R: aaa tgc aga cgc agg gta ca
*Matrix metalloproteases 1 (MMP 1)*	F: agg tct ctg agg gtc aag caR: ctg gtt gaa aag cat gag ca
*Matrix metalloproteases 3 (MMP 3)*	F: tgc ttt gtc ctt tga tgc tg R: aag ctt cct gag gga ttt gc
α*-smooth muscle actin (ACTA2)*	F: ctg agc gtg gct att cct tc R: ttc tca agg gag gat gag ga

### Western blotting

The expression of keratocan, lumican and decorin proteoglycans, ALDH1 and ALDH3 crystallins, carbohydrate sulfotransferase 6 (CHST6), Collagen Type I and V, MMP1 and MMP9 proteases, fibronectin and α-smooth muscle actin (αSMA) were analyzed from day 30 compressed collagen gels embedded with keratocytes in both RA-treated and control groups lysates using ice-cold RIPA lysis buffer supplemented with 1x Proteases Inhibitors Cocktail (Roche, 04693116001) for 10 minutes. Both human donor cornea and keratocytes grown for 7 d in serum-containing medium (bFBS) were used as controls. After precipitation with 4 × volumes of ethanol and pellet resuspension in sample buffer, lysates were run by reducing SDS-PAGE using 10% Mini-Protean precast gels (Bio-Rad, 4561034) and blotted onto polyvinylidene difluoride (PVDF) membranes (Thermo Scientific, 88518). Membranes were then blocked in Tris-buffered saline (Fisher Scientific, 10103203) supplemented with 5% bovine serum albumin (First-Link, 40–00–450) and 0.1% Tween 20 (Fisher Scientific, 10246910), and incubated with primary antibodies against keratocan (Santa Cruz Biotechnology, sc-66941), lumican (kindly given by Dr Bruce Caterson, Cardiff School of Biosciences, Cardiff, UK), decorin (CalBiochem, PC673), ALDH1A1, MMP1, MMP9, CHST6 (Abcam, ab23375, EP1247Y, EP1254, ab154332 respectively), ALDH3A1 (Thermo Scientific, 10304480), αSMA and fibronectin (Vector Labs, VPS281 and VPF705, respectively) diluted 1:500 in blocking solution, followed by corresponding horseradish peroxidise-conjugated secondary antibodies. Anti-tubulin was used for protein loading normalization. Quantification was performed by densitometry analysis of imaged bands using Image J v1.46 (National Institute of Health, Bethesda, MD).

### Immunofluorescence staining

Corneal constructs were imaged by immunofluorescence microscopy. Briefly, 7 µm sections of the gels were cut at −20°C using a cryotome (Leica Microsystems, Wetzlar, Germany) and collected on adhesion glass slides (Thermo Scientific, 640-ADH-006). Sections were fixed in 4% paraformaldehyde for 20 minutes prior to incubation with 2% goat serum (Sigma-Aldrich, G9023) and 2% bovine serum albumin (First-Link) at room temperature to block non-specific binding. Sections were then incubated overnight at 4°C with primary antibodies against keratocan, lumican, ALDH1, Collagen type I and V (same as used for Western blotting). Incubation with secondary antibodies was carried out for 1 hour using Alexa Fluor® 488-conjugated goat anti-mouse or anti-rabbit (1:200, Life Technologies, A11001 and A11008 respectively). Finally, the slides were imaged by fluorescence microscopy (Carl Zeiss Meditec, Germany).

### Data analysis and statistics

Error bars represent the SD of the mean. Differences between groups were determined using Student's *t*-test. Significance between groups was established for *p* < 0.05, denoted as ‘*’ within figures.

## CONCLUSION

The greatest challenge in developing a bio-engineered cornea has always been to recapitulate the biological corneal stroma within the artificial scaffold. This study demonstrates that RA can improve the quality of 3D collagen gels as an *in vitro* model of the corneal stroma. Furthermore, our results move the potential use of RA a step forward suggesting that RA could be used clinically. As RA supplementation induced keratocytes within a 3D collagen gel matrix to increase proteoglycans production, particularly keratocan and lumican, and remain non-contractile, it is possible that RA may help reduce scarring following wound healing by promoting proliferation and replacement with normal keratocytes within corneal stroma without the need for fibroblast/myofibroblast transformation that can alter proteoglycan and collagen production and lead to corneal haziness.
